# Bonnesen-style inequality for the first eigenvalue on a complete surface of constant curvature

**DOI:** 10.1186/s13660-017-1462-4

**Published:** 2017-08-15

**Authors:** Niufa Fang, Jiazu Zhou

**Affiliations:** 1 School of Mathematics and Statistics, Southwest University, Chongqing, 400715 China; 2 College of Science, Wuhan University of Science and Technology, Wuhan, Hubei 563006 China

**Keywords:** 53A25, 53A10, 53C23, the first eigenvalue, Cheeger’s isoperimetric constants, Bonnesen-style inequality

## Abstract

By Cheeger’s isoperimetric constants, some lower bounds and upper bounds of $\lambda_{1}$, the first eigenvalue on a complete surface of constant curvature, are given. Some Bonnesen-style inequalities and reverse Bonnesen-style inequalities for the first eigenvalue are obtained. Those Bonnesen-style inequalities obtained are stronger than the well-known Osserman’s results and the upper bound is stronger than Osserman’s results (Osserman in Proceedings of the International Congress of Mathematicians, Helsinki, 1978).

## Introduction

The classical isoperimetric problem is to determine a plane figure of largest possible area whose boundary has a specific length and it was known in Ancient Greece. However, the first mathematically rigorous proof was obtained only in the nineteenth century and it was well recognized by Weierstrass though Bernoulli, Euler and Lagrange once claimed the proof that was found flawed later. Hurwitz published a short proof using the Fourier series that applies to arbitrary plane domain *D* whose boundary *∂D* was not assumed to be smooth. An elegant direct proof, based on the comparison of a smooth simple closed curve with a circle, was given by Schmidt in 1938 by using only the arc length formula, expression for the area of a plane region from Green’s theorem, and the Cauchy-Schwarz inequality [2]. Many other proofs have been found and some of them were stunningly simple. The isoperimetric problem has been extended in multiple ways, for example, to domains on surfaces and in higher dimensional spaces, or more generally to integral currents and analytic manifolds, but the proof is too difficult.

Let *D* be a domain (subset with nonempty interiors) in the Euclidean plane $\mathbb{R}^{2}$ with the boundary composing of the simple curve of length *L* and area *A*. Then

1.1 $$\begin{aligned} L^{2}-4\pi A\geq0, \end{aligned}$$

the equality holds when and only when *D* is a disc.

It is known that the isoperimetric inequality (1.1) is equivalent to the following Sobolev inequality (see [3]):

If *f* has compact support in $D\subset\mathbb{R}^{2}$, then

1.2 $$\begin{aligned} \biggl( \int_{D}|\nabla f| \biggr)^{2}-4\pi \int_{D}f^{2}\geq0. \end{aligned}$$

Here ∇ denotes the gradient operator. The equality holds in (1.2) if and only if *f* is the characteristic function of balls.

During 1920s, Bonnesen proved a series of inequalities of the form

1.3 $$\begin{aligned} L^{2}-4\pi A\geq B_{D}, \end{aligned}$$

where the quantity $B_{D}$ on the right-hand side is a non-negative geometric invariant of significance and vanishes only when *D* is a disc.

An inequality of the form (1.3) is called the Bonnesen-style inequality, and it is stronger than the classical isoperimetric inequality. The Bonnesen-style inequality has been extended to surfaces of constant curvature and higher dimensions and many Bonnesen-style inequalities have been found during the past. Mathematicians are still working on unknown Bonnesen-style inequalities of geometric significance [3–6]. The isoperimetric inequality for domains on surfaces *M* of constant curvature can be stated as follows.

Let *D* be a compact domain on the surface *M* of constant curvature. Let *A* and *L* denote the area and the boundary length of *D*, respectively. Then

$$\begin{aligned} L^{2}-4\pi A+MA^{2}\geq0, \end{aligned}$$

the equality holds if and only if *D* is a geodesic disc.

The Bonnesen-style inequality for domains on surfaces of constant curvature was first investigated by Santaló [7, 8]. Klain obtained some new Bonnesen-style inequalities for domains on surfaces of constant curvature [9]. By the kinematic formulas in integral geometry, Xu, Zhou et al. also obtained Bonnesen-style inequalities on a complete surface of constant curvature (see [10, 11]). Osserman [5] studied the Bonnesen-style inequality for the domains on surfaces with the bounded Gauss curvature. More Bonnesen-style homothetic (Wulff) inequalities were obtained in [4, 12, 13]. Another important extension of the isoperimetric problem in analysis is eigenvalues of the Laplacian.

*Eigenvalues of the Laplacian*. Let *D* be a domain with smooth boundary *∂D* on a compact Riemannian surface *M*. The eigenvalue problem

$$\begin{aligned}& \Delta u+\lambda u = 0 \quad \text{in } D; \\& u|_{\partial D} = 0, \end{aligned}$$

is known to have a complete system of eigenfunctions $u=\phi_{n}$, with corresponding eigenvalues $\lambda_{n}$, where

$$0=\lambda_{0}< \lambda_{1}< \lambda_{2}< \cdots< \lambda_{p}< \cdots\nearrow\infty. $$

One would ask naturally a basic question: how are the properties of domain *D* on a compact Riemannian surface *M*, that is, area of $D\subset M$, length and integrals of curvature of *∂D*, reflected in the set of eigenvalues $\{\lambda_{n}\}$?

In this paper, we will investigate the Bonnesen-style inequalities for the first eigenvalue $\lambda_{1}$ of Laplacian on the complete surface. Let *M* be a compact Riemannian surface and Δ be the Laplacian-Beltrami operator acting on differential functions $C^{\infty}(M)$. It is known that Δ is an elliptic operator. The first eigenvalue $\lambda_{1}$ on domain $D\subset M$ can be also characterized by [1]

1.4 $$\begin{aligned} \lambda_{1}(D)=\inf_{f\in\mathfrak{F}} \frac{\int_{D}|\nabla f|^{2}}{\int _{D}|f|^{2}}, \end{aligned}$$

where $\mathfrak{F}$ is the set of smooth functions in *D* vanishing on the boundary.

The Laplace operator on a Riemannian manifold, its spectral theory and the relations between its first eigenvalue and the geometrical data of the manifold, such as curvatures, diameter, injectivity radius and volume, have been extensively studied in the recent mathematical literature. Amazing connections between the isoperimetric inequality and the first eigenvalue of Laplacian operator have been found during the past decades. One of the basic results is that Cheeger connected the first eigenvalue $\lambda_{1}$ of the Laplacian on a manifold to certain isoperimetric constants. For a domain *D* on a two-dimensional surface, Cheeger considers the quantity

1.5 $$\begin{aligned} h=\inf_{D'\subset F}\frac{L'}{A'}, \end{aligned}$$

where *F* is the family of relatively compact subdomains of *D*, $A'$ and $L'$ are the area and the boundary length of subdomain $D'\subset D$, respectively. Cheeger proved that

1.6 $$\begin{aligned} \lambda_{1}(D)\geq\frac{1}{4}h^{2}. \end{aligned}$$

The upper estimate of the first eigenvalue of Laplacian has been discussed by geometers and analysts. Hersch [14] obtained an upper bound for manifolds homeomorphic to the two sphere. Cheeger [15], Chavel and Feldman [16] obtained upper bound for manifolds with non-negative Ricci curvature. The comparison theorem of Cheng [17] gives a sharp upper bound for general Riemannian manifold in terms of the Ricci curvature and the diameter of domain.

While the progress has been made on the upper bound, not too much is known about the lower bound of the first eigenvalue. The best result is due to Lichnerowicz [18] who gives a computable sharp lower bound for manifolds whose Ricci curvature is bounded from below by a positive constant. Cheeger [19] also gives a lower estimate for general manifolds in terms of some isoperimetric constants. These constants of Cheeger, however, are not computable. Cheng [20] observed that if the manifold is a two-dimensional convex surface, then the isoperimetric constant has a lower bound in terms of the diameter. Since 1979, Li and Yau have been trying to obtain the lower bound of the first eigenvalue [21, 22]. Chen introduced the method in probability theory to improve almost all results proved by others in [23]. For more detailed isoperimetric properties and the first eigenvalue, one can refer to [1, 18, 24–31].

In [5], Osserman considered the first eigenvalue $\lambda_{1}$ on the two-dimensional manifolds with bounded Gauss curvature and obtained some lower and upper bounds of the first eigenvalue by using Cheeger’s isoperimetric constant as follows:

Let *S* be a compact simply connected surface with Gauss curvature *K*, $K\leq-\alpha^{2}$, $\alpha>0$. If $D\subset S$ is simply connected and *ρ* is inradius of *D*, then

1.7 $$\begin{aligned} \lambda_{1}(D)-\frac{\alpha^{2}}{4}\geq\frac{\alpha^{2}}{4} \operatorname{csch}^{2} \alpha\rho. \end{aligned}$$

In this paper, we obtain the following lower bound of the first eigenvalue that is stronger than Osserman’s result (1.7):

*Let* *S* *be a simply connected complete surface with Gauss curvature* $K\leq0$ *everywhere. For any simply connected domain* $D\subset S$ *, let* *ρ* *denote its inradius and* *R* *denote its circumradius. Let*

$$\begin{aligned} \alpha^{2}=\inf_{D}(-K),\qquad \beta^{2}=\sup_{D}(-K), \quad 0< \alpha\leq\beta, \end{aligned}$$

*then*

$$\begin{aligned} \lambda_{1}(D)-\frac{\alpha^{2}}{4}\geq\frac{\alpha^{2}}{4} \operatorname{csch}^{2} \alpha\rho+B, \end{aligned}$$

*where the quantity* *B* *is a positive number depending on* *α,* *β,* *ρ,* *R.*

We also obtain the upper bound of the first eigenvalue. By Cheng’s eigenvalue comparison theorem ([17], Theorem 1.1), we obtain a stronger upper bound of the first eigenvalue $\lambda_{1}$ (Theorem 4.1).

## The Bonnesen-style isoperimetric inequalities

Let $D_{r}^{M} $ denote the geodesic disc of radius *r* on the complete simply connected surface of constant Gauss curvature $K\equiv M$. Let $A_{r}^{M}, L_{r}^{M} $ be, respectively, the area and the length of boundary of $D_{r}^{M}$. Then the explicit expressions for these quantities are

$$\begin{aligned}& M=-\alpha^{2} < 0\mbox{:}\quad L_{r}^{M}= 2\pi \frac{\sinh\alpha r}{\alpha},\qquad A_{r}^{M}= 4\pi\frac{\sinh^{2}\frac{\alpha r}{2}}{\alpha^{2}}=2 \pi\frac {\cosh\alpha r -1}{\alpha^{2}}; \\& M=0\mbox{:}\quad L_{r}^{M}=2\pi r,\qquad A_{r}^{M}= \pi r^{2}; \\& M=\alpha^{2} >0\mbox{:}\quad L_{r}^{M}= 2\pi \frac{\sin\alpha r}{\alpha},\qquad A_{r}^{M}= 4\pi\frac{\sin^{2}\frac{\alpha r}{2}}{\alpha^{2}}=2 \pi\frac {1-\cos\alpha r }{\alpha^{2}}. \end{aligned}$$

For the geodesic disc, the following equation can be easily verified in all three cases:

2.1 $$ \bigl(L_{r}^{M}\bigr)^{2}-4\pi A_{r}^{M}+M \bigl(A_{r}^{M} \bigr)^{2}=0. $$

The isoperimetric inequality on a surface of constant curvature $K\equiv M$ is

2.2 $$ L^{2}-4\pi A+MA^{2}\geq0. $$

Namely, given a domain *D* of area *A*, if *r* is chosen so that $A_{r}^{M}$ equals *A*, then (2.1) and (2.2) imply $L\geq L_{r}^{M}$, so that the disc $D_{r}^{M}$ has minimum boundary length among all domains of the same area.

Osserman considered the isoperimetric inequality of two-dimensional complete surface with bounded Gauss curvature [5].

Let *D* be a simply connected domain whose Gauss curvature *K* satisfies $K\leq M$. Let *L* and *A* be the boundary length and the area of *D*, respectively. Then

2.3 $$\begin{aligned} L^{2}-4\pi A+MA^{2}\geq0, \end{aligned}$$

where the equality holds if and only if $K\equiv M$ and *D* is a geodesic disc.

Osserman also obtained the following Bonnesen-style isoperimetric inequalities.

### Theorem A

[5]

*Let* *D* *be a simply connected domain whose Gauss curvature* *K* *satisfies* $K\leq M$. *Let* *ρ* *be the inradius of* *D*, *A* *be the area of* *D* *and* *L* *be the length of its boundary*. *Then the following inequalities are equivalent*:

2.4 $$\begin{aligned}& LL_{\rho}^{M}+MAA_{\rho}^{M} \geq 2\pi \bigl(A+A_{\rho}^{M}\bigr), \end{aligned}$$

2.5 $$\begin{aligned}& L^{2}-4\pi A +MA^{2} \geq \biggl(L-\frac{L_{\rho}^{M}}{A_{\rho}^{M}}A \biggr)^{2}, \end{aligned}$$

2.6 $$\begin{aligned}& L^{2}-4\pi A +MA^{2} \geq \bigl(L-L_{\rho}^{M} \bigr)^{2}+M\bigl(A-A_{\rho}^{M}\bigr)^{2}. \end{aligned}$$

*Moreover*, *if* $MA<4\pi$, *then these inequalities are equivalent to*

2.7 $$ L^{2}-4\pi A +MA^{2} \geq \biggl( \frac{2\pi}{L_{\rho}^{M}}\bigl(A-A_{\rho}^{M}\bigr) \biggr)^{2}. $$

Osserman estimated lower bounds of the first eigenvalue by Cheeger’s isoperimetric constants as follows.

### Theorem B

[5]

*Let* *S* *be a simply connected complete surface with Gauss curvature* *K*, $K\leq-\alpha^{2}$, $\alpha>0$. *Then*, *for any domain* $D\subset S$ *of circumradius* *R*,

2.8 $$\begin{aligned} \lambda_{1}(D)-\frac{\alpha^{2}}{4}\geq\frac{\alpha^{2}}{4}( \operatorname{csch} \alpha R )^{2}. \end{aligned}$$

*If* *D* *is simply connected and* *ρ* *is its inradius*, *then*

2.9 $$\begin{aligned} \lambda_{1}(D)-\frac{\alpha^{2}}{4}\geq\frac{\alpha^{2}}{4}( \operatorname{csch} \alpha \rho) ^{2}. \end{aligned}$$

## The lower bound of $\lambda_{1}$

In this section, we give some lower bounds of the first eigenvalue $\lambda_{1}$ by Cheeger’s isoperimetric constants and Bonnesen-style isoperimetric inequalities. We need the following lemmas.

### Lemma 3.1

*Let* $f(r)$ *be continuously differentiable on the interval* $0\leq r\leq r_{0}$. *Suppose that*, *except at a finite number of the points in the interval*, $f''(r)$ *exists and satisfies*

3.1 $$\begin{aligned} f''(r)+cf(r)\leq0, \qquad f(0)=0, \qquad f'(0)=a \end{aligned}$$

*for some constants* *a*, *c*. *Let* $h(r)$ *be the unique solution of*

3.2 $$\begin{aligned} h''(r)+ch(r)= 0, \qquad h(0)=0, \qquad h'(0)=1. \end{aligned}$$

*Let* *s* *be any number such that* $h(r)>0$ *for* $0< r< s$, *and let* $r_{1}=\min\{r_{0}, s\}$. *Then*

3.3 $$\begin{aligned} f(r)\leq ah(r) \end{aligned}$$

*for* $0\leq r\leq r_{1}$.

### Proof

Let $\phi(r)=\frac{f(r)}{h(r)}$. Then by (3.1) and (3.2)

$$\begin{aligned} \bigl(h^{2}\phi'\bigr)'= \bigl(f'h-fh'\bigr)'=f''h-fh'' \leq0, \end{aligned}$$

except at the singular points. By the mean value theorem, $h^{2}\phi'$ is a weakly monotone decreasing function, and hence

$$\begin{aligned} \bigl(h^{2}\phi'\bigr) (r)\leq \bigl(h^{2}\phi'\bigr) (0)=0, \quad 0\leq r\leq r_{1}. \end{aligned}$$

That is, $\phi'(r)\leq0$, and hence

$$\begin{aligned} \frac{f(r)}{h(r)}= \phi(r)\leq\phi(0)=\lim_{r\rightarrow 0} \frac{f(r)}{h(r)}=a \end{aligned}$$

for $0\leq r\leq r_{1}$. □

### Lemma 3.2

*Let* $D_{\rho}$ *be a geodesic disc of radius* *ρ*, *and let* $A_{\rho}$ *be the area of* $D_{\rho}$. *If* $M\leq K\leq0$ *on* $D_{\rho}$, *then*

3.4 $$ A_{\rho} \leq A_{\rho}^{M}, $$

*where equality holds if and only if* $K\equiv M$ *on* $D_{\rho}$.

### Proof

We introduce geodesic polar coordinates in $D_{\rho}$. The metric can be written as $ds^{2}=dr^{2}+g(r,\theta)\, d\theta^{2}$, where for each *θ*, the function $f(r)=\sqrt{g}(r,\theta)$ satisfies $f(0)=0$, $f'(0)=1$. Since $K\leq 0$, the geodesic disc of radius *ρ* always exists. Then with the fact $K=-\frac{1}{\sqrt{g}}\frac{\partial^{2}}{\partial r^{2}}\sqrt{g}$ and the condition $M\leq K\leq0$, $f(r)$ satisfies (3.1), with $a=1$, $c=M$, $r_{0}=\rho$. By (3.3), we have

3.5 $$\begin{aligned} A_{\rho}= \int_{0}^{2\pi} \int_{0}^{\rho}\sqrt{g}(r,\theta)\, dr\, d\theta \leq2 \pi \int_{0}^{\rho}h(r)\, dr=A_{\rho}^{M}. \end{aligned}$$

Since $h(r)$ can be written explicitly as $h(r)=\frac{1}{2\pi}L_{\rho}^{M}$, it satisfies (3.2) and $A_{\rho}=\int_{0}^{\rho}L(r)\,dr$. The equality holds if and only if $\sqrt{g}(r,\theta)\equiv h(r)$, hence $K\equiv M$. □

### Theorem 3.1

*Let* *S* *be a simply connected complete surface with Gauss curvature* $K\leq0$ *everywhere*. *For any simply connected domain* $D\subset S$, *let* *A*, *L*, *R* *be the area*, *the boundary length and the circumradius of* *D*, *respectively*. *Let*

3.6 $$\begin{aligned} \alpha^{2}=\inf_{D}(-K), \qquad \beta^{2}=\sup_{D}(-K), \quad 0< \alpha\leq\beta, \end{aligned}$$

*then*

3.7 $$\begin{aligned} \lambda_{1}(D)-\frac{\alpha^{2}}{4}\geq\frac{\beta^{2}}{4} \biggl(\operatorname{csch} \frac{\beta R}{2} \biggr)^{2}. \end{aligned}$$

### Proof

By Lemma 3.2 and (3.6), we have

3.8 $$\begin{aligned} A_{R}^{-\beta^{2}}\geq A_{R}\geq A. \end{aligned}$$

Via (3.6), the isoperimetric inequality (2.3) can be rewritten as

$$\begin{aligned} \biggl(\frac{L}{A} \biggr)^{2}\geq4\pi \frac{1}{A}+\alpha^{2}. \end{aligned}$$

Then, by (3.8), we have

3.9 $$\begin{aligned} \biggl(\frac{L}{A} \biggr)^{2} \geq& 4\pi\frac{1}{A}+ \alpha^{2} \\ \geq&4\pi\frac{1}{A_{R}^{-\beta^{2}}}+\alpha^{2} \\ =&\beta^{2} \biggl(\operatorname{csch} \frac{\beta R}{2} \biggr)^{2} + \alpha^{2} . \end{aligned}$$

By (1.6) and (3.9), then

$$\begin{aligned} \lambda_{1}(D)-\frac{\alpha^{2}}{4}\geq\frac{\beta^{2}}{4} \biggl(\operatorname{csch} \frac{\beta R}{2} \biggr)^{2}. \end{aligned}$$

We complete the proof. □

Since $\frac{x}{\sinh x}$ is monotonically decreasing for $x\geq 0$, hence (3.7) is stronger than (2.8) if $\frac{1}{2} \beta < \alpha$. By (2.4) we obtain a lower bound of $\lambda_{1}$ that is stronger than the one in (2.9).

### Theorem 3.2

*Let* *S* *be a simply connected complete surface with Gauss curvature* $K\leq0$ *everywhere*. *For any simply connected domain* $D\subset S$, *let* *A*, *L*, *ρ*, *R* *be the area*, *the boundary length*, *the inradius and the circumradius of* *D*, *respectively*. *Let*

3.10 $$\begin{aligned} \alpha^{2}=\inf_{D}(-K), \qquad \beta^{2}=\sup_{D}(-K), \quad 0< \alpha\leq\beta, \end{aligned}$$

*then*

3.11 $$\begin{aligned} \lambda_{1}(D)-\frac{\alpha^{2}}{4}\geq\frac{\alpha^{2}}{4} ( \operatorname{csch} \alpha\rho)^{2}+B, \end{aligned}$$

*where*

$$B=\frac{1}{4} \biggl(\frac{\beta\sinh\frac{\alpha\rho}{2}}{\alpha\sinh \frac{\beta R}{2}} \biggr)^{4} \biggl( \frac{\alpha}{\sinh\alpha\rho} \biggr)^{2} +\frac{1}{2} \biggl( \frac{\beta\sinh\frac{\alpha\rho}{2}}{\alpha\sinh\frac{\beta R}{2}} \biggr)^{2} \frac{\alpha^{2}\coth\alpha\rho}{\sinh\alpha\rho}. $$

### Proof

By (2.4) and (3.10), we have

$$\begin{aligned} \frac{L}{A}L_{\rho}^{-\alpha^{2}}\geq2\pi \biggl(1+ \frac{A_{\rho}^{-\alpha ^{2}}}{A} \biggr)+\alpha^{2}A_{\rho}^{-\alpha^{2}}, \end{aligned}$$

and hence,

3.12 $$\begin{aligned} \frac{L}{A}\geq\frac{A_{\rho}^{-\alpha^{2}}}{A}\frac{\alpha}{\sinh \alpha\rho}+\alpha \coth\alpha\rho. \end{aligned}$$

By (3.4) and (3.10), (3.12) can be rewritten as

$$\begin{aligned} \frac{L}{A} \geq& \frac{A_{\rho}^{-\alpha^{2}}}{A}\frac{\alpha}{\sinh\alpha\rho}+\alpha \coth\alpha \rho \\ \geq&\frac{A_{\rho}^{-\alpha^{2}}}{A_{R}^{-\beta^{2}}}\frac{\alpha}{\sinh \alpha\rho}+\alpha\coth\alpha\rho \\ =& \biggl(\frac{\beta\sinh\frac{\alpha\rho}{2}}{\alpha\sinh \frac{\beta R}{2}} \biggr)^{2}\frac{\alpha}{\sinh\alpha\rho}+\alpha \coth\alpha\rho. \end{aligned}$$

By (1.5) and (1.6), we have

$$\begin{aligned} \lambda_{1}(D) \geq&\frac{1}{4} \biggl\{ \biggl( \frac{\beta\sinh \frac{\alpha\rho}{2}}{\alpha\sinh\frac{\beta R}{2}} \biggr)^{2}\frac{\alpha}{\sinh\alpha\rho}+\alpha\coth \alpha\rho \biggr\} ^{2} \\ =&\frac{\alpha^{2}}{4}(\coth\alpha\rho)^{2}+B \\ =&\frac{\alpha^{2}}{4}+\frac{\alpha^{2}}{4}(\operatorname{csch} \alpha\rho )^{2}+B, \end{aligned}$$

and

$$B=\frac{1}{4} \biggl(\frac{\beta\sinh\frac{\alpha\rho}{2}}{\alpha\sinh \frac{\beta R}{2}} \biggr)^{4} \biggl( \frac{\alpha}{\sinh\alpha\rho} \biggr)^{2}+\frac {1}{2} \biggl( \frac{\beta\sinh \frac{\alpha\rho}{2}}{\alpha\sinh\frac{\beta R}{2}} \biggr)^{2}\frac{\alpha^{2}\coth\alpha\rho}{\sinh\alpha\rho}. $$

 □

Since $B\geq0$, hence inequality (3.11) is stronger than inequality (2.9). Let $R=\rho$ in Theorem 3.2, that is, let *D* be a geodesic disc with radius *ρ* on *S*. Then let $\rho\rightarrow\infty$ in (3.11), we immediately obtain the following.

### Corollary 3.1

*Let* *S* *be a simply connected complete surface with* $K\leq-\alpha^{2}$, $\alpha>0$ *everywhere*, *then*

$$\begin{aligned} \lambda_{1}(S)\geq\frac{\alpha^{2}}{4}. \end{aligned}$$

Next, we give a lower bound of the first eigenvalue $\lambda_{1}$.

### Theorem 3.3

*Let* *D* *be a complete simply connected surface and* *A* *denote the area of* *D*. *Then*

$$\begin{aligned} \lambda_{1}(D)\geq\frac{4\pi}{A}. \end{aligned}$$

### Proof

By Sobolev inequality (1.2) and Hölder’s inequality, we have

$$\begin{aligned} 4\pi \int_{D}f^{2}\, dx \leq& \biggl( \int_{D}|\nabla f|\, dx \biggr)^{2} \\ \leq& \int_{D}1^{2}\, dx \int_{D}|\nabla f|^{2}\,dx \\ =&A \int_{D}|\nabla f|^{2}\, dx, \end{aligned}$$

that is,

$$\begin{aligned} \frac{\int_{D}|\nabla f|^{2}\, dx}{\int_{D} f^{2}\, dx}\geq\frac{4\pi}{A}. \end{aligned}$$

By (1.4), we have

$$\begin{aligned} \lambda_{1}=\inf_{f\neq0}\frac{\int_{D}|\nabla f|^{2}\, dx}{\int_{D} f^{2}\, dx}\geq \frac{4\pi}{A}. \end{aligned}$$

 □

By Lemma 3.2, we obtain a lower bound of $\lambda_{1}$.

### Corollary 3.2

*Let* *S* *be a simply connected complete surface with Gauss curvature* *K*, $-\beta^{2}\leq K\leq0$ *everywhere*. *Suppose* $D\subset S$, *then*

$$\begin{aligned} \lambda_{1}(D)\geq\beta^{2} \biggl(\operatorname{csch} \frac{\beta R}{2} \biggr)^{2}. \end{aligned}$$

*Here* *R* *is the circumradius of* *D*.

## The upper bound of $\lambda_{1}$

In this section, we consider the upper bound of the first eigenvalue $\lambda_{1}$. We start with the following eigenvalue comparison theorem proved by Cheng in [17]. Denote the open geodesic ball of radius *r* with center *x* by $D(x;r)$. Denote by $V_{n}(M;r)$ the geodesic ball of radius *r* in the *n*-dimensional simply connected space form with constant sectional curvature *M*. We write $\lambda_{1}(\overline{D(x;r)})$ as $\lambda_{1}(D(x;r))$.

### Theorem C

*Suppose that* *S* *is a complete Riemannian manifold and Ricci curvature of* $S\geq(n-1)M$, $n=\dim S$. *Then*, *for* $x\in S$, *we have*

$$\begin{aligned} \lambda_{1}\bigl(D(x;r)\bigr)\leq\lambda_{1} \bigl(V_{n}(M;r)\bigr) \end{aligned}$$

*and equality holds if and only if* $D(x;r)$ *is isometric to* $V_{n}(M;r)$.

In particular, the eigenvalue comparison theorem is also valid when *S* is a two-dimensional complete simply connected surface.

### Corollary 4.1

*Suppose that* *S* *is a complete simply connected surface with Gauss curvature* $K\geq M$. *Let* $D_{r}$ *be a geodesic disc with radius* *r* *on* *S*, *then*

$$\begin{aligned} \lambda_{1}(D_{r})\leq\lambda_{1} \bigl(D_{r}^{M}\bigr), \end{aligned}$$

*where equality holds if and only if* $K\equiv M$ *on* *S*.

The next lemma will be needed in proving our theorem.

### Lemma 4.1

[5]

*Suppose that* *S* *is a simply connected complete surface with Gauss curvature* $K\leq0$ *everywhere*. *Let* $D_{\rho}$ *be a geodesic disc of radius* *ρ*. *If*

4.1 $$\begin{aligned} \alpha^{2}=\inf_{D_{\rho}}(-K), \qquad \beta^{2}=\sup_{D_{\rho}}(-K), \quad 0< \alpha\leq\beta, \end{aligned}$$

*then*

4.2 $$\begin{aligned} \lambda_{1}(D_{\rho})\leq \biggl( \frac{\beta^{2}}{2\alpha\coth\alpha \rho} \biggr)^{2}+ \biggl(\frac{\pi}{\rho} \biggr)^{2}. \end{aligned}$$

Combining Corollary 4.1 and Lemma 4.1 immediately yields the following.

### Theorem 4.1

*Suppose that* *S* *is a simply connected complete surface with Gauss curvature* *K*, $-\beta^{2}\leq K\leq0$ *everywhere*. *Let* $D_{\rho}$ *be a geodesic disc of radius* *ρ*, *then*

4.3 $$\begin{aligned} \lambda_{1}(D_{\rho})\leq \biggl( \frac{\beta}{2\coth\beta \rho} \biggr)^{2}+ \biggl(\frac{\pi}{\rho} \biggr)^{2}. \end{aligned}$$

### Proof

Since $D_{\rho}^{-\beta^{2}}$ satisfies the hypotheses of Lemma 4.1 when $\alpha=\beta$, hence

$$\begin{aligned} \lambda_{1}\bigl(D_{\rho}^{-\beta^{2}}\bigr)\leq \biggl( \frac{\beta}{2\coth\beta \rho} \biggr)^{2}+ \biggl(\frac{\pi}{\rho} \biggr)^{2}. \end{aligned}$$

By Corollary 4.1, we immediately obtain (4.3). We complete the proof of Theorem 4.1. □

Since the function $x\coth x$ is monotonically increasing for $x\geq0$, hence inequality (4.3) is stronger than (4.2). Let $\rho\rightarrow\infty$ in (4.3), we can easily have the following corollary.

### Corollary 4.2

*Let* *S* *be a simply connected complete surface with Gauss curvature* *K*, $-\beta^{2}\leq K\leq0$ *everywhere*. *Then*

$$\begin{aligned} \lambda_{1}(S)\leq\frac{\beta^{2}}{4}. \end{aligned}$$
